# Exploring the Multifactorial Regulation of PIEZO1 in Chondrocytes: Mechanisms and Implications

**DOI:** 10.7150/ijms.111082

**Published:** 2025-07-24

**Authors:** Gongwu Yuan, Ziren Xiong, Xi Ke, Guodong Wang, Ximing Liu, Zhigang Li

**Affiliations:** 1 Department of Orthopedic Surgery, Hubei Provincial Hospital of Integrated Chinese & Western Medicine, Wuhan 430015, China.; 2 Department of Orthopedics, General Hospital of Central Theater Command, Wuhan 430070, China.; 3 College of Acupuncture and Orthopedics, Hubei University of Chinese Medicine, Wuhan 430065, China; 4 School of Medicine, Wuhan University of Science and Technology, Wuhan 430081, China.

**Keywords:** chondrocyte, mechanotransduction, mechanosensitive ion channels, PIEZO1

## Abstract

The mechanosensitive PIEZO1 ion channel plays a pivotal role in the regulation of chondrocyte function and is involved in various physiological and pathological processes, including cartilage degradation and osteoarthritis (OA). This review explores the regulatory mechanisms governing PIEZO1 activation and its interactions with mechanical stress, extracellular matrix (ECM) stiffness, inflammatory factors, and other ion channels. We discuss the role of PIEZO1 in calcium signaling, its modulation by ECM stiffness, and the implications for cartilage health, particularly under high mechanical load or inflammatory conditions. Additionally, the review highlights pharmacological modulators, including PIEZO1 activators such as Yoda1 and Jedi1/2, and inhibitors like GsMTx4, as potential therapeutic targets for OA treatment. The synergistic interactions of PIEZO1 with other mechanosensitive channels, such as TRPV4 and PIEZO2, are also examined, providing insights into their collective role in mechanotransduction. Further research is essential to clarify the spatiotemporal activation patterns of PIEZO1, its downstream effects, and the potential for targeting this channel in clinical interventions for degenerative cartilage diseases.

## 1. Introduction

Chondrocytes, the only cell type in articular cartilage, play a pivotal role in regulating joint mechanical stress distribution, sensing external stimuli, and maintaining the metabolic homeostasis of the extracellular matrix (ECM) under mechanical load 1-3. These cells are able to perceive mechanical load through mechanosensitive ion channels, including PIEZO1, PIEZO2, and transient receptor potential vanilloid 4 (TRPV4) 4. The mechanical stress that is exerted on the cells is a primary physical regulatory factor that influences a number of cellular processes, including proliferation, differentiation, apoptosis, and matrix synthesis or degradation. Under normal physiological conditions, moderate mechanical stress promotes ECM synthesis, such as type II collagen and aggrecan, thereby maintaining cartilage function 5,6. However, excessive or abnormal mechanical loads (e.g., high pressure or shear force) have been shown to induce apoptosis of the cartilage cells, as well as inflammatory responses and matrix degradation, thus leading to cartilage degeneration and the subsequent development of osteoarthritis (OA) and post-traumatic osteoarthritis (PTOA) 7-8. Conversely, a reduction in mechanical load has been shown to alleviate cartilage destruction, subchondral bone changes, and secondary inflammatory alterations in OA animal models 9.

In recent years, the critical role of mechanosensitive ion channels (MSCs) in chondrocyte mechanotransduction has become increasingly evident. The influx of extracellular calcium is a central mechanism underlying cellular responses to mechanical load 10. Variations in the concentration of calcium ions within the cell are primarily mediated by calcium channels located on the cell membrane. Chondrocytes express a number of different calcium channels, including TRPV4, T/L-type voltage-gated Ca²⁺ channels (VGCCs), PIEZO1/2 channels, transient receptor potential (TRP) family channels, and calcium release-activated Ca²⁺ channels (CRACs) 11. Among these, PIEZO1—a large mechanosensitive calcium channel—responds directly to membrane tension changes, triggering calcium ion (Ca²⁺) influx and initiating a cascade of intracellular signaling pathways 12. This process regulates the proliferation, apoptosis, and autophagy of cells, as well as the metabolic balance of the ECM 13.

PIEZO1, the largest plasma membrane ion channel complex identified to date, exhibits unique structural and functional characteristics that are central to cellular responses to mechanical stimuli. Unlike other ion channels or proteins, PIEZO1 is a high-molecular-weight protein that lacks significant sequence similarity with known ion channels or proteins, thus marking it as a distinct mechanosensitive channel 14. Structurally, PIEZO1 shares similarities with PIEZO2, being large conserved transmembrane proteins composed of homotrimeric structures resembling a triskelion or three-bladed propeller 14-16. This distinctive architecture enables PIEZO1 to sense mechanical forces by responding to membrane tension changes, opening ion channels for calcium influx, and subsequently activating intracellular signaling cascades.

The structure of PIEZO1 can be divided into two primary modules: a peripheral mechanotransduction module and a central ion-conduction pore module 15. The peripheral mechanotransduction module, comprising multiple subunits, transduces mechanical stimuli into chemical signals through transmembrane structures. Meanwhile, the central ion-conduction pore module forms a functional ion channel, facilitating transmembrane ion flux, particularly that of calcium ions, which are essential for channel activation 17. The trimeric structure of PIEZO1 features three peripheral blades, each consisting of 14 distinct transmembrane regions, which are critical for its mechanosensing capabilities 18. These blades enable PIEZO1 to detect external mechanical stimuli via membrane deformation, resulting in ion flux modulation. Furthermore, the C-terminal region of PIEZO1 contains two transmembrane structures, which are thought to form the ion-conduction pore 19. While the structural details of PIEZO1 have been extensively elucidated in murine models, further studies are required to clarify its configuration in humans. The complexity and uniqueness of PIEZO1 make it a vital model for studying mechanosensitive ion channels, providing essential insights into its role in cellular mechanotransduction.

Significant progress has been made in elucidating the physiological and pathological roles of PIEZO1 in chondrocytes, thereby highlighting its growing importance in cartilage biology and the progression of related diseases. Studies have revealed that PIEZO1 plays critical roles in OA and PTOA 20, as well as in bone fracture healing, skeletal development 21, cartilage regeneration, and other mechanically related diseases 22-27. However, most current research has focused on PIEZO1 activation under single mechanical stress modalities, with limited exploration of its differential regulation under diverse mechanical stimuli, such as tension, shear, and compression 1,28. Moreover, the intricate interactions and feedback mechanisms between PIEZO1 and inflammatory factors (e.g., IL-1β) remain poorly understood. For instance, IL-1β upregulates PIEZO1 expression through the NF-κB signaling pathway, while abnormal activation of PIEZO1 has been found to exacerbate inflammatory responses 29. Furthermore, research on pharmacological and chemical regulation of PIEZO1 remains in its infancy, with only a few activators (e.g., Yoda1) and inhibitors (e.g., GsMTx4) identified, whose specificity and translational potential require further investigation 30-32.

This review aims to systematically summarize the factors influencing PIEZO1 channels in chondrocytes, including mechanical stress, matrix environment, inflammatory factors, chemical substances, and other mechanosensitive channels. Additionally, it explores their regulatory roles and molecular mechanisms in related diseases, thus providing both theoretical support and practical insights for the advancement of PIEZO1 research and the development of therapeutic targets.

## 2. Mechanical Stress and Piezo1 in Chondrocytes

PIEZO1 is one of the key ion channels in chondrocytes responsible for responding to mechanical stress. Its activation leads to changes in intracellular calcium concentrations, thereby influencing the physiological functions of chondrocytes. PIEZO1 is able to sense and respond to diverse forms of mechanical stimuli, including externally applied poke, stretch, and shear stress, as well as endogenously derived local membrane tension and myosin II-mediated traction forces 12,33. The role of mechanical stress in maintaining the function of these cells is twofold. Moderate mechanical stimulation activates PIEZO1, contributing to calcium homeostasis, sustaining chondrocyte function, and promoting ECM synthesis. Conversely, abnormal or excessive mechanical stress has been shown to trigger excessive calcium influx, resulting in cellular dysfunction, damage, and apoptosis 1,7,28. Therefore, the type, intensity, and mode of mechanical stress significantly influence PIEZO1 activation and chondrocyte physiological responses.

### 2.1 Physiological Mechanical Stress and Piezo1 Activation

Under physiological conditions, chondrocytes are exposed to moderate mechanical stress, such as during natural joint movement and weight-bearing. These stimuli appropriately activate PIEZO1, leading to calcium signaling that maintains cellular homeostasis. Physiological mechanical stress supports the synthesis of ECM proteins, including type II collagen and aggrecan, which are essential for preserving cartilage structure and function 5,6.

Moderate mechanical stress promotes ECM synthesis in chondrocytes via PIEZO1 activation, in addition to regulating various functions of the cells, including proliferation, differentiation, and metabolic activities 34. It has been demonstrated that appropriate mechanical loading upregulates PIEZO1 expression, thereby enhancing the cell's ability to respond to mechanical signals. Conversely, insufficient mechanical loading significantly reduces PIEZO1 expression in chondrocytes, impairing their capacity to sense and respond to mechanical stimuli. The study further elucidates that the downregulation of PIEZO1, induced by reduced mechanical loading, activates the ApoE signaling pathway, which in turn induces cellular senescence in the cells. The upregulation of senescence markers such as p16 and p21 is a hallmark of these conditions, further exacerbating cellular senescence and functional decline. In animal models, reduced mechanical loading diminishes PIEZO1 expression, disrupts cartilage proliferation and differentiation, impedes callus formation, and hinders endochondral ossification, ultimately delaying fracture healing 35.

The loss of PIEZO1 has been demonstrated lead to functional disorders in chondrocytes. In healthy joints, the mechanosensing function of these cells is characterised by high levels of organisation and regulation, which ensure the production of adaptive cellular responses to varying stress environments 2. PIEZO1 deficiency has been shown to disrupt calcium signaling pathways, leading to functional abnormalities in these cells, characterised by increased expression of matrix-degrading genes such as matrix metalloproteinases (MMPs). This, in turn, has been demonstrated to exacerbate cellular aging and dysfunction 21. Studies indicate that moderate mechanical stress not only activates PIEZO1 but also works synergistically with other mechanosensitive ion channels, such as TRPV4, to promote proliferation of the cells responsible for producing the cartilage in the joint 20. This interplay between multiple ion channels plays a crucial role in physiological adaptation and mitigating OA progression.

### 2.2 Pathological Stress and Piezo1 Activation

Pathological mechanical stress, such as excessive high-intensity loads or traumatic stresses, leads to overactivation of PIEZO1 channels, resulting in excessive intracellular calcium accumulation and promoting chondrocyte apoptosis 11,36,37. This process activates multiple intracellular signaling pathways, including calcium/calmodulin-dependent protein kinase II (CaMKII) and mitogen-activated protein kinase (MAPK), which culminate in apoptosis or senescence 38,39. Overactivation has been observed to upregulate proteins associated with cell cycle arrest, including Kif18A and β-tubulin, which ultimately impedes chondrocyte proliferation 40. Additionally, excessive mechanical loading has been demonstrated to accelerate the process of chondrocyte senescence via the activation of PIEZO1. *In vivo* studies using chondrocyte-specific Piezo1 knockout mice (Col2a1-Cre; Piezo1^fl/fl^) demonstrated that excessive mechanical loading activates PIEZO1 in chondrocytes, leading to increased intracellular calcium accumulation, which in turn induces senescence and accelerates cartilage degeneration 41. In spinal growth plate chondrocytes, PIEZO1 activation triggers calcium overload and apoptosis under compression 42. Whether this mechanism operates in articular chondrocytes requires further validation. Notably, pathological mechanical stress not only elevates intracellular calcium levels but also induces oxidative stress, further damaging chondrocytes 43. PIEZO1 activation is a critical trigger in this process 38. Consequently, the inhibition of aberrant PIEZO1 activation may represent a potential strategy for mitigating OA progression caused by pathological mechanical stress.

### 2.3 Types and Intensities of Stress on Piezo1

The effects of different types and intensities of mechanical stress on PIEZO1 activation and its biological outcomes are distinct. For instance, stretch stress, compressive stress, and shear stress all activate PIEZO1, but the mechanisms and effects of these different stresses vary 44,45.

Stretch stress activates PIEZO1 by inducing membrane deformation, promoting calcium influx, and regulating ECM metabolism and cytoskeletal remodeling. It is particularly critical for type II collagen synthesis, enhancing cartilage resilience and function 11. However, excessive stretch stress, such as high-frequency cyclic stretching, significantly increases intracellular calcium levels, exacerbates ECM degradation through F-actin polymerization, and elevates reactive oxygen species (ROS), leading to MMP-13 expression and subsequent ECM breakdown and apoptosis 46.

Chondrocytes experience compressive stress during weight-bearing activities. Moderate compressive stress activates PIEZO1, promoting calcium influx and enhancing ECM synthesis (e.g., type II collagen and aggrecan) whilst suppressing MMP expression, thereby maintaining homeostasis within the cell 47. However, it has been demonstrated that sustained or excessive compressive stress can lead to overactivation of PIEZO1, resulting in excessive calcium influx, cellular senescence, apoptosis, and matrix degradation, disrupting adaptive responses and contributing to cartilage degeneration and OA progression 13,32,48,49.

Excessive shear stress has been demonstrated to potentially compromise the functionality of chondrocytes, particularly under pathological conditions such as arthritis. Research indicates that mechanical stress conditions (e.g., pressure, duration) significantly influence PIEZO1 activation. For example, Li et al. (2017) reported that mechanical loading at 150 kPa promoted survival of cartilage cells, while loading at 200 kPa significantly increased cell death and cartilage degeneration 50. Similarly, Chen et al. (2007) observed that chondrocyte proliferation remained unaffected by a 90 kPa load applied for 1 hour had no effect on cartilage cell proliferation, but that a 6-hour stimulus significantly reduced proliferation 51. In addition, studies on PIEZO1 activation thresholds provide insights into its biophysical responses. Savadipour et al. found that PIEZO1 responds to membrane strain only when membrane tension reaches a specific threshold (apparent strain ~1.31), triggering calcium signaling 47 These findings reveal that the biophysical response of chondrocytes to mechanical stress exhibits a strong threshold dependence, suggesting that the impact of mechanical stress of varying intensities on PIEZO1 activation may demonstrate pronounced nonlinear characteristics.

PIEZO1's mechanosensitivity stems from its unique trimeric propeller-like structure, which directly senses membrane tension changes. Structural studies reveal that mechanical forces induce conformational rearrangements in PIEZO1's peripheral blades, leading to pore dilation and Ca²⁺ influx 18. Physiological mechanical stress (e.g., 10-15% strain) promotes transient Ca²⁺ oscillations that activate Ca²⁺/calmodulin-dependent kinase II (CaMKII), enhancing ECM synthesis via SOX9 and COL2A1 upregulation 47,48. Conversely, pathological overload (>20% strain) triggers sustained Ca²⁺ overload, activating MAPK/p38 and NF-κB pathways to drive MMP-13 expression and apoptosis 13,38. Notably, shear stress amplifies PIEZO1-TRPV4 crosstalk via cytoskeletal remodeling, exacerbating IL-6/IL-8 release through NLRP3 inflammasome activation 34,48 These findings highlight the strain threshold-dependent dual roles of PIEZO1 in cartilage homeostasis and degeneration.

Despite the considerable progress that has been made in comprehending the function of PIEZO1 in chondrocytes, the majority of studies have been confined to examining a single type of mechanical stress (e.g., stretch or compression). Comparative analyses of multiple mechanical stimuli, such as shear and cyclic stress, remain scarce. Furthermore, while physiological and pathological mechanical stresses differentially regulate PIEZO1 activation, precise modulation of stress types, intensities, and activation levels remains a key challenge. Consequently, future research should emphasize the dynamic spatiotemporal regulation of PIEZO1 under diverse stress conditions to enable precise control of its activity and avoid cell damage due to overactivation.

## 3. Matrix Environment and Piezo1 Regulation

Chondrocytes adjust their metabolism, differentiation, and phenotype maintenance by sensing ECM stiffness. Research indicates that ECM stiffness influences PIEZO1 activation, modulating intracellular calcium signaling and regulating the physiological and pathological responses of chondrocytes 52.

### 3.1 Matrix Stiffness and Piezo1 Activation

ECM stiffness is a crucial physical factor affecting chondrocyte function and mechanical adaptability. In low-stiffness ECM environments, the phenotype of these cells is maintained at a relatively stable level, as evidenced by the high expression levels of type II collagen and aggrecan, which are essential for the normal structure and function of cartilage 53. Conversely, in conditions of high ECM stiffness, PIEZO1 activation is repressed, thereby supporting homeostasis and physiological functions in these cells. In contrast, high ECM stiffness has been shown to suppress phenotype maintenance in these cells, promote cytoskeletal remodeling, and enhance focal adhesion formation, thus resulting in heightened PIEZO1 activation. This, in turn, causes significant increases in intracellular calcium concentration 37. Calcium, a key secondary messenger, regulates vital biological processes such as metabolism, differentiation, and autophagy. However, excessive calcium signaling in high-stiffness ECM environments may drive ECM degradation and induce disc chondrocyte senescence and apoptosis through oxidative and endoplasmic reticulum stress pathways 54.

The present study sought to investigate the impact of ECM stiffness on PIEZO1 activation and calcium signaling. While low ECM stiffness permits PIEZO1 to primarily mediate calcium signaling for maintaining normal chondrocyte functions and phenotypes, high ECM stiffness amplifies calcium oscillations, intensifying chondrocyte responses to mechanical stress 55. It has been demonstrated that high ECM stiffness not only increases calcium influx but also exacerbates oxidative stress and endoplasmic reticulum stress, accelerating cellular senescence and apoptosis. Conversely, softer ECM environments have been shown to inhibit PIEZO1 activation, thereby maintaining intracellular calcium homeostasis, reducing ECM degradation, and preventing cartilage damage and aging 54.

However, the extent to which ECM stiffness affects PIEZO1 localization and function in chondrocytes remains to be elucidated, beyond the regulation of PIEZO1 activity. As with other cell types, changes in ECM properties may influence PIEZO1 redistribution on the chondrocyte membrane, though this remains to be confirmed. For instance, studies suggest that cytoskeletal remodeling and increased focal adhesion formation in response to high ECM stiffness may drive PIEZO1 accumulation on the cell surface, enhancing its activation levels.

### 3.2 Primary Cilia and PIEZO1 in chondrocytes

Primary cilia, organelles located on the surface of chondrocytes, have been shown to play a vital role in sensing mechanical signals and modulating cellular responses 56. It has been demonstrated that primary cilia are capable of regulating PIEZO1 sensitivity, thereby altering intracellular calcium levels 57. Furthermore, the ECM stiffness has been observed to modulate PIEZO1 activation, and also to influence primary cilia morphology by altering the activity of histone deacetylase 6 (HDAC6) 58. The loss of primary cilia has been associated with significant changes in cartilage structure and mechanical properties 59, as well as abnormalities in weight-bearing cartilage. This emphasises the pivotal interplay between ECM stiffness, PIEZO1 channels, and primary cilia in chondrocyte adaptive responses to varying mechanical environments. Primary cilia act as a bridge between ECM stiffness and PIEZO1 activity, further modulating cellular responses to changes in the mechanical environment. These findings reveal a complex interaction between ECM stiffness, PIEZO1, and primary cilia, contributing to the molecular mechanisms underlying chondrocyte adaptation to ECM hardness.

In environments where the ECM has low stiffness, homeostasis of the chondrocyte is supported by the inhibition of excessive PIEZO1 activation, with a resultant reduction of ECM degradation and cellular damage. Conversely, elevated ECM stiffness has been demonstrated to result in overactivation of PIEZO1, enhanced calcium signaling, and accelerated cellular senescence and apoptosis, which may contribute to the progression of degenerative cartilage diseases. While current studies have elucidated the relationship between ECM stiffness and PIEZO1, further research is required to determine PIEZO1 activation thresholds under varying stiffness levels and its cooperative interaction with other ion channels, such as TRPV4. For example, evidence suggests that PIEZO1 dominates calcium signaling in low-stiffness ECM, whereas TRPV4 plays a more significant role in high-stiffness ECM 37 Additionally, ECM stiffness appears to regulate PIEZO1 localization and cytoskeletal interactions, offering novel molecular mechanisms for how chondrocytes sense and respond to ECM hardness. Further research is needed to clarify the spatiotemporal characteristics of PIEZO1 activation, its interplay with the cytoskeleton and primary cilia, and its precise regulatory mechanisms under different mechanical environments.

### 3.3 Lipid Regulation of PIEZO1 Channels

The lipid bilayer actively modulates PIEZO1 activity through composition and mechanical properties. Cholesterol-rich lipid rafts enhance PIEZO1 clustering and stabilize its open state under mechanical stress, while cholesterol depletion reduces channel activation 60,61. Phosphatidylserine (PS) externalization in stressed membranes amplifies PIEZO1 sensitivity, particularly in inflammatory cartilage, exacerbating calcium overload and ECM degradation 62. Additionally, phosphatidylinositol 4,5-bisphosphate (PIP2) binds PIEZO1's C-terminal domain, facilitating channel clustering; PIP2 depletion disrupts mechanotransduction even under high load 19. These lipid-protein interactions highlight the membrane's dynamic role in PIEZO1 regulation 63,64.

In osteoarthritic cartilage, altered lipid metabolism—elevated cholesterol oxides and ceramides—disrupts membrane fluidity, driving aberrant PIEZO1 activation and calcium dysregulation 65,66. This lipid imbalance correlates with cartilage degradation and oxidative stress, suggesting therapeutic targeting of lipid-PIEZO1 interactions 54. Strategies such as cholesterol modulation or PIP2 stabilization may mitigate OA progression by restoring mechanotransduction homeostasis.

High ECM stiffness (> 20 kPa) enhances integrin-β1 clustering and RhoA/ROCK-mediated actomyosin contractility, increasing membrane tension to sensitize PIEZO1 37,67. This primes chondrocytes for exaggerated Ca²⁺ responses to mechanical stimuli, accelerating senescence via oxidative stress 43. Conversely, soft ECM (< 5 kPa) suppresses PIEZO1 by reducing focal adhesion kinase (FAK) signaling, favoring YAP/TAZ nuclear retention to maintain chondrogenic phenotypes 53. Primary cilia act as mechanosensory hubs: HDAC6 deacetylates α-tubulin under high stiffness, shortening cilia length and disrupting PIEZO1-TRPV4 colocalization, thereby dysregulating Ca²⁺ homeostasis 20,58. This ECM-cytoskeleton-cilia axis critically determines PIEZO1's mechanoadaptive responses.

## 4. Inflammatory Factors and Piezo1 Regulation

Chondrocytes have been shown to express functional interleukin-1 (IL-1) receptors and respond to both isoforms of IL-1 (α and β) 68. These cells are able to sense and react to inflammatory factors, particularly interleukin-1 (IL-1) and tumour necrosis factor-alpha (TNF-α). The secretion of matrix-degrading enzymes by these cells has been shown to be induced by inflammatory factors, including IL-1β, IL-1α, and TNF-α, thereby exacerbating cartilage degeneration. This process is further facilitated by the modulation of PIEZO1 channel expression and function. Recent studies reveal that mechanical stress-induced PIEZO1 activation triggers mitochondrial Ca²⁺ overload and mitochondrial DNA (mtDNA) release, which activates the cGAS-STING pathway, amplifying inflammatory responses through type I interferon signaling 69. This process plays a crucial role in the progression of diseases like OA.

### 4.1 Inflammatory Modulation of Piezo1 Expression

IL-1β, a primary inflammatory mediator, has been demonstrated to upregulate PIEZO1 expression in chondrocytes 29. Furthermore, PIEZO1 activation has been shown to be positively correlated with the release of inflammatory cytokines such as IL-1β in the nucleus 70. The influence of IL-1β has been found to result in a significant increase in both the mRNA and protein levels of PIEZO1. This upregulation may occur through the activation of the nuclear factor-kappa B (NF-κB) signaling pathway, as inhibiting the Ca²⁺/NF-κB pathway substantially reduces PIEZO1-dependent inflammatory responses 70. Mechanistically, mechanical stress amplifies this regulation by inducing Piezo1-mediated Ca²⁺ influx, which activates NF-κB and synergizes with IL-1β to promote mtDNA release and cGAS-STING signaling 69.

IL-1β-induced PIEZO1 activation partially suppresses autophagy in chondrocytes while promoting apoptosis. This is evidenced by increased expression of apoptosis-related proteins such as cleaved caspase-3 and Bax, and decreased levels of autophagy-related proteins like Bcl2, LC3, and p62. Inhibition of the PI3K/AKT/mTOR pathway has been shown to mitigate the suppression of autophagy and activation of apoptosis caused by PIEZO1, suggesting it as a potential therapeutic target for PIEZO1 regulation 29. Furthermore, IL-1α has been observed to enhance PIEZO1 expression through pathways involving p38 MAP kinase, hepatocyte nuclear factor 4 (HNF4), and ATF2/CREBP1. CREBP1 binds directly to the proximal promoter of the PIEZO1 gene, promoting protein synthesis and enhancing cellular sensitivity to mechanical stress 48. The structural and functional similarities between IL-1β and IL-1α further support IL-1β's regulatory role in the activity of PIEZO1 in chondrocyte. PIEZO1 activation correlated closely with the expression of apoptosis-related proteins such as Bax and caspase-3, suggesting that the inhibition of PIEZO1 expression may effectively alleviate IL-1β-induced damage to chondrocytes 29.

### 4.2 Feedback Between Inflammation and Piezo1 Activation

Inflammatory factors have been demonstrated to exacerbate damage to chondrocytes through the activation of PIEZO1 channels. Furthermore, these factors have been shown to form positive feedback loops that worsen the progression of OA. For instance, the PIEZO1 agonist Yoda1 significantly increases the expression of inflammatory cytokines such as IL-6 and IL-8, while PIEZO1 knockdown partially reverses this effect 1 Notably, mechanical stress-induced PIEZO1 activation promotes mitochondrial permeability transition pore (mPTP) opening via Ca²⁺ overload, leading to mtDNA leakage into the cytoplasm. Cytosolic mtDNA activates the cGAS-STING pathway, which drives the production of pro-inflammatory cytokines (e.g., IL-6, TNF-α, IFN-β) and exacerbates cartilage degradation 69. This pathway synergizes with NLRP3 inflammasome activation, as PIEZO1 activation also promotes caspase-1-dependent IL-1β maturation 30, creating a dual inflammatory cascade. The activation of PIEZO1 has been shown to be closely associated with the inflammatory response, with IL-1β, IL-6, and IL-8 levels having been found to be positively correlated with its activity. Moreover, PIEZO1 activation has been shown to promote the assembly of the NLRP3 inflammasome, significantly upregulate caspase-1 activity, and facilitate the maturation of IL-1β 30. This process is further amplified by cGAS-STING activation, which induces TBK1/IRF3 phosphorylation and type I interferon production, establishing a feedforward loop between mechanical stress and inflammation 69.

Additionally, PIEZO1 activity can be indirectly regulated by inflammatory factors. For instance, elevated joint osmotic pressure has been observed to activate PIEZO1 in chondrocytes, inducing calcium overload, endoplasmic reticulum, and mitochondrial stress, and leading to apoptosis. Chondrocyte damage has the effect of triggering interstitial fluid leakage and inflammatory infiltration (e.g., TNF-α, prostaglandin E2), which in turn damage newly formed cartilage tissue. This exudate increases intra-articular pressure, which in turn activates PIEZO1, thus creating a cycle of injury 71. The interplay between inflammatory factors and mechanical stress exacerbates cartilage degeneration and OA progression, thus providing new insights for therapeutic strategies.

While the role of PIEZO1 activation in inflammatory cartilage injury has been preliminarily elucidated, the specific mechanisms by which different inflammatory factors (e.g., TNF-α, IL-1β, IL-6) regulate PIEZO1 remain incompletely understood, particularly in various physiological and pathological contexts. Moreover, the interaction between inflammatory factors and mechanical stress in modulating PIEZO1 activity and its effects on apoptosis and matrix degradation warrants further investigation. A more complete understanding of the precise role of PIEZO1 in inflammatory joint diseases, such as OA, and the refinement of its regulation, could offer novel therapeutic approaches for clinical applications.

### 4.3 Inflammatory Signaling Converges on PIEZO1 via Transcriptional and Post-Translational Regulation

IL-1β binds IL-1R to activate NF-κB, which directly binds the PIEZO1 promoter to upregulate its expression 29. Concurrently, TNF-α induces mitochondrial ROS, oxidizing PIEZO1's cysteine residues (Cys1563/Cys1707) to enhance channel open probability 48. This ROS-NF-κB-PIEZO1 feedforward loop sustains inflammatory ECM degradation. Furthermore, IL-1β suppresses autophagy via PI3K/AKT/mTOR, impairing PIEZO1 turnover and prolonging Ca²⁺ signaling 29. Pharmacologically, GsMTx4 disrupts this cycle by inhibiting PIEZO1/calcineurin/NFAT1 signaling, restoring autophagy flux and reducing apoptosis 32.

## 5. Chemical and Pharmacological Modulation of Piezo1

The recent discovery of PIEZO1 channels has prompted a surge in research interest, driven by their critical roles across multiple systems and tissues. Consequently, there has been a proliferation of studies investigating the development of PIEZO1 activators and inhibitors. These substances have emerged as valuable tools for modulating PIEZO1 activity and exploring their therapeutic potential.

### 5.1 Activators of Piezo1

Yoda1, first identified in 2015 by the Coste team, is one of the most well-known PIEZO1 activators in heterologous systems 30. It has been demonstrated that Yoda1 can promote conformational changes in PIEZO1, thereby stabilizing its open state and lowering the mechanical threshold required for channel activation 72. Acting as a molecular wedge, Yoda1 enhances calcium influx by altering PIEZO1's configuration. In addition, studies have shown that Yoda1 treatment significantly reduces the proliferation and colony formation capabilities of primary chondrocytes while increasing cellular senescence. This effect is attributed to the acceleration of intracellular calcium accumulation, which in turn promotes the process of cellular senescence 73. Furthermore, Yoda1 has been observed to upregulate the expression of MMPs and inflammatory cytokines such as IL-6 and IL-8, exacerbating ECM degradation 1,34. In another study, Yoda1 was observed to reduce the expression of Col10a1 in ATDC5 cells, thus implicating its role in ECM disruption 24. Despite these effects, Yoda1 has shown potential in promoting chondrocyte differentiation and improving fracture healing under conditions of mechanical unloading, thus highlighting its dual roles 35. Its ability to activate PIEZO1 without the need for external mechanical stimuli makes it a valuable tool for exploring PIEZO1 regulation.

Jedi1/2, a class of PIEZO1 activators identified in 2018 by Xiao and He, represents a novel category of pharmacological agents. In contrast to Yoda1, Jedi1/2 exerts its effect by targeting PIEZO1's peripheral blades, thereby modulating the activity of its lever-like structures composed of blades and beams 74. In a manner analogous to Yoda1, Jedi1/2 has the capacity to activate PIEZO1 in the absence of mechanical stimuli, and further derivatives, such as Yoda2, have been synthesised to enhance PIEZO1 efficacy 75. However, the application of Jedi1/2 and Yoda2 in chondrocytes has not yet been explored, providing future directions for research on novel PIEZO1 activators.

### 5.2 Inhibitors of Piezo1

While PIEZO1 channels have been shown to play critical roles in various physiological and pathological processes. However, overactivation of these channels has been implicated in diseases such as cartilage degeneration and OA. Consequently, PIEZO1 inhibitors have gained attention as a means of regulating channel activity. Existing PIEZO1 inhibitors, including GsMTx4, gadolinium ions (Gd³⁺), ruthenium red, and drugs like benzbromarone, are primarily nonspecific and act through diverse mechanisms 31,76.

GsMTx4, a 34-amino acid peptide derived from tarantula venom, is one of the most commonly used PIEZO1 inhibitors. It has been demonstrated that GsMTx4 exerts its inhibitory effect by reducing PIEZO1 activation, rather than by direct binding to the channel itself 31. By penetrating deeper into the lipid bilayer under increased membrane tension, GsMTx4 decreases lateral pressure on the membrane, ultimately inhibiting PIEZO1 activity 77. Experimental evidence has demonstrated that GsMTx4 significantly attenuates calcium influx induced by mechanical stress, thereby mitigating the responses of articular cells to damaging strains 78. In cartilage explants, GsMTx4 pre-treatment has been shown to reduce mechanical stress-induced damage 36. *In vivo*, GsMTx4 has been observed to alleviate cartilage degeneration and pain by inhibiting the PIEZO1/Calcineurin/NFAT1 signaling axis, reducing calcium influx, and preserving proteoglycan integrity 7,32,42,79. These findings position GsMTx4 as a promising tool for treating degenerative diseases like OA, though its nonspecific action may limit its therapeutic precision.

As nonspecific cation channel blockers, Gd³⁺ and ruthenium red have been shown to effectively inhibit PIEZO1 activity by disrupting membrane mechanosensing capabilities 12,80,81 These inhibitors reduce calcium influx by impairing PIEZO1 activation. However, their lack of specificity limits their clinical utility, as they may interfere with other ion channels. A similar inhibitory effect has been demonstrated for Streptomycin, a PIEZO1 open conformation inhibitor, but this approach faces similar challenges 31,82.

Although GsMTx4 and other PIEZO1 inhibitors show potential in mitigating mechanical stress-induced damage to chondrocytes, inflammation, and ECM degradation, their nonspecific effects on other mechanosensitive ion channels may impact therapeutic outcomes. It is therefore recommended that future research prioritize the development of more specific PIEZO1 inhibitors, with a view to minimising off-target effects. Furthermore, there is a necessity for additional studies to be conducted on the safety and efficacy of PIEZO1 inhibitors in clinical applications.

The employment of PIEZO1 activators and inhibitors has resulted in the development of novel molecular instruments, facilitating the exploration of this channel's functions in both physiological and pathological contexts. Activators such as Yoda1 and Jedi1/2 facilitate the study of PIEZO1's activation mechanisms, while inhibitors such as GsMTx4 and Dooku1 hold therapeutic promise for mitigating conditions associated with PIEZO1 overactivation. However, further investigation is required into the specificity, safety, and clinical utility of these substances, particularly in the context of cartilage degeneration and OA treatment.

### 5.3 Natural Compounds and Piezo1 Regulation

In recent years, natural compounds and TCM monomers have gained attention for their potential in regulating PIEZO1 channels. Compounds such as artemisinin and urocortin have demonstrated efficacy in modulating PIEZO1 activation, cartilage cell function, and associated diseases, thus providing a novel therapeutic approach for degenerative conditions such as OA.

Artemisinin, a naturally occurring compound derived from *Artemisia annua*, has been identified as a modulator of PIEZO1 activity. In chondrocytes, Artemisinin was found to inhibit mechanical stress-induced PIEZO1 activation, thereby reducing intracellular calcium accumulation, and alleviating cellular stress responses and ECM degradation 83. Its mechanism of action involves suppressing PIEZO1 activity to protect against damage related to OA. Studies have shown that artemisinin reduces calcium influx triggered by PIEZO1 activation and modulates downstream PI3K/AKT signaling pathways, improving cartilage erosion, proteoglycan loss, synovial hyperplasia, osteophyte formation, and pain in OA mouse models. Additionally, artemisinin treatment has been shown to reduce the expression of RUNX2, ADAMTS5, and MMP13 while increasing COL2A1 expression, indicating a protective effect against PIEZO1-mediated cartilage damage 83. Collectively, these findings suggest artemisinin's potential in controlling excessive PIEZO1 activation.

Urocortin exerts an indirect inhibitory effect on PIEZO1 activity by binding to corticotropin-releasing factor receptor 1 (CRF-R1). This interaction activates adenylyl cyclase, increasing cyclic AMP (cAMP) production, and inactivates phospholipase A2 (PLA2), thereby reducing membrane phospholipid degradation and maintaining PIEZO1 in a closed state 84. Research on cartilage explants subjected to excessive impact loading has shown that urocortin effectively blocks PIEZO1 activation, significantly reducing chondrocyte damage and cartilage degeneration 85. The ability of urocortin to regulate membrane phospholipid metabolism provides a valuable foundation for the development of new cartilage-protective agents.

TMAO, a gut microbial metabolite derived from diets rich in quaternary amines and meat, has been linked to the progression of OA 86. Higher concentrations of TMAO have been observed to correlate with increased synovial inflammation scores in patients with OA 87. Zhuang et al. have demonstrated that TMAO significantly upregulates PIEZO1 mRNA and protein expression *in vitro* and *in vivo*, increasing intracellular calcium levels and sensitizing chondrocytes to mechanical loads 88. This heightened sensitivity exacerbates mechanical strain-induced chondrocyte apoptosis and OA-related joint damage. While TMAO has been shown to enhance PIEZO1 expression, it does not appear to directly activate the channel. Consequently, dietary modifications aimed at reducing TMAO production may offer a potential strategy for mitigating PIEZO1 overactivation.

The G-protein-coupled estrogen receptor (GPER) has been demonstrated to play a key role in regulating PIEZO1 function in chondrocytes. GPER activation has been shown to promote the translocation of the transcription factor Yes-associated protein (YAP) from the cytoplasm to the nucleus, thereby modulating apoptosis through the interaction with the Hippo signaling pathway, involving the proteins Yes-associated protein 29 (YAP), Rho-associated protein kinase (ROCK), LIMK and cofilin. In mechanical stress-induced PIEZO1 activation, GPER has been identified as a crucial regulator. Its expression has been shown to be significantly reduced in human OA cartilage, and intra-articular injection of a GPER-selective agonist has been observed to alleviate cartilage degeneration in OA animal models 38. The protective effects of GPER may be particularly relevant in female OA patients due to its association with oestrogen levels 89. Furthermore, the absence of PIEZO1 has been demonstrated to enhance GPER expression at both the mRNA and protein levels, thereby underscoring their interplay in response to mechanical loads.

Yoda1 binds PIEZO1's C-terminal extracellular cap, stabilizing the open state by reducing the mechanical activation threshold 72. Jedi1/2, in contrast, targets the peripheral blades to allosterically gate the channel 74. Conversely, GsMTx4 embeds into lipid bilayers under high membrane tension, counteracting lateral pressure to mechanically “clamp” PIEZO1 77. Natural compounds like artemisinin block PIEZO1 via direct pore occlusion, while urocortin indirectly inhibits it via CRF-R1/cAMP/PLA2 signaling to reduce membrane phospholipid degradation 83,84. These structure-activity relationships underscore the potential for designing domain-specific therapeutics.

Natural compounds like artemisinin, urocortin, TMAO, and GPER significantly regulate PIEZO1 channels, especially in cartilage degeneration and OA. Artemisinin inhibits calcium influx, reducing cellular stress and ECM degradation; urocortin stabilizes membrane tension via CRF-R1, keeping PIEZO1 closed; TMAO upregulates PIEZO1 expression, sensitizing chondrocytes to mechanical stress and promoting cartilage degradation. However, their low bioavailability and metabolic instability, particularly for artemisinin, limit clinical applications. Given the complex regulatory mechanisms of PIEZO1, combining these compounds with established therapies like anti-inflammatory medications may offer more effective OA treatment by addressing multiple aspects of PIEZO1 modulation. Future research should explore these interactions to enhance therapeutic outcomes in OA and other PIEZO1-related conditions.

## 6. Interactions Between Piezo1 and Other Mechanosensitive Channels

### 6.1 TRPV4 and Piezo1 Interaction

TRPV4 channel is another key mechanosensitive ion channel in chondrocytes that responds to extracellular mechanical stimuli by promoting calcium influx 90. TRPV4 and PIEZO1 channels exhibit synergistic activity in regulating mechanical signal transduction, mediating compression-induced membrane strain, and resulting in intracellular calcium oscillations 20,91. Furthermore, they cooperate in responding to harmful mechanical loads, contributing to the mechanotransduction in chondrocytes 78,90.

However, TRPV4 and PIEZO1 differ in their mechanogating mechanisms. Unlike PIEZO1, TRPV4 cannot be efficiently gated by pressure-induced membrane stretching and is not activated by cell indentation 20. While TRPV4 inhibition has shown protective effects against aging-related OA, it does not prevent trauma-induced OA, underscoring its specific role in mechanotransduction 92. TRPV4 is known to respond primarily to low-intensity mechanical stress, initiating calcium signalling during physiological strains of between 3% and 8%. In contrast, PIEZO1 exhibits higher sensitivity to high-intensity strains, such as those caused by traumatic mechanical stress of up to 18% 78. This divergence in response thresholds gives rise to a complementary signaling network between TRPV4 and PIEZO1 20. Furthermore, Studies have revealed that physiological mechanical loading transduced by TRPV4 and supraphysiological loading transduced by PIEZO1 elicit distinct transcriptomic responses 93. TRPV4 modulates cytoskeletal tension, indirectly affecting PIEZO1 activation and mechanical adaptability 20.

The interaction between TRPV4 and PIEZO1 extends to their regulation by exogenous stimuli, with both channels being targeted by their respective activators (e.g., Yoda1 for PIEZO1 and GSK1016790A for TRPV4). The effects of the first agonist applied are suppressed by the second, highlighting a regulatory interplay 34. In addition, the dual silencing of TRPV4 and PIEZO1 genes has been shown to enhance the expression of critical ECM components like aggrecan and type II collagen, suggesting that their combined activity plays an essential role in cartilage repair 94.

A central question in understanding TRPV4-PIEZO1 synergy is whether TRPV4 directly modulates PIEZO1 activity through interactions on the cell membrane. Evidence suggests that TRPV4 activation can alter the open state of PIEZO1 channels, thereby modifying calcium signaling characteristics within chondrocytes. This interplay has significant implications for how these cells adapt to complex mechanical environments and provides a foundation for developing therapies that simultaneously target TRPV4 and PIEZO1 to optimise mechanotransduction and cartilage protection. The coordinated activity of TRPV4 and PIEZO1 channels plays a vital role in the mechanotransduction process within chondrocytes. While TRPV4 responds to low-intensity stress and PIEZO1 to high-intensity stress, their interactions create a dynamic signaling network that is essential for adapting to varying mechanical environments. Further research into the mutual modulation mechanisms between these channels could pave the way for innovative therapeutic strategies targeting both TRPV4 and PIEZO1, especially in the treatment of OA and other cartilage-related diseases.

### 6.2 Piezo2 Synergy with Piezo1

PIEZO1 and PIEZO2 are two critical mechanosensitive ion channels expressed across various tissues and cells, each with distinct physiological and pathological roles. PIEZO1 is ubiquitously expressed, while PIEZO2 is predominantly found in peripheral sensory neurons, where it is involved in tactile sensation, pain perception, and proprioception 95,96. Interestingly, PIEZO2 has also been identified in chondrocytes, suggesting a functional role in cartilage physiology 20,78. Both channels exhibit voltage-dependent inactivation 97, with PIEZO2 inactivating more rapidly than PIEZO1. For instance, under a -80 mV holding potential, PIEZO2 has an inactivation time constant of approximately 5 ms, compared to PIEZO1's 15 ms 12,98.

The synergy between PIEZO1 and PIEZO2 in chondrocytes plays a crucial role in the perception and transduction of mechanical signals. Collectively, their activation regulates the chondrocyte response to mechanical stress, particularly in bone and cartilage tissues. The channels facilitate calcium signaling, which in turn modulates downstream transcription factors such as NFAT, YAP1 and β-catenin 78,99. PIEZO2 primarily mediates calcium signaling under low-intensity stress, thereby maintaining mechanical sensitivity within a physiological range, whereas PIEZO1 takes on a more dominant role under high-intensity stress 100. he functional interdependence of these channels is further highlighted by the observation that single knockouts of PIEZO1 or PIEZO2 nearly abolish mechanically induced calcium influx in chondrocytes 78. This finding suggests that their roles in mechanotransduction are complementary, with both channels jointly mediating the response to harmful mechanical stimuli 36. However, under certain conditions, PIEZO1 can operate independently of PIEZO2, particularly during high-intensity strain where PIEZO1 alone can initiate calcium signaling 47.

Despite the demonstrated synergy between PIEZO1 and PIEZO2, recent studies have indicated that the dual knockout of these channels does not significantly affect joint development or normal function. In a Gdf5-Cre transgenic mouse model, PIEZO1 and PIEZO2 double-knockout mice exhibited OA progression similar to that of the control group, suggesting that the direct influence of Piezo channels on OA progression may be limited 101. This finding implies a potential functional redundancy between PIEZO1 and PIEZO2 in chondrocytes, with less pronounced synergistic effects under physiological and pathological conditions than had previously been anticipate.

While PIEZO2 enhances PIEZO1 sensitivity and the two channels jointly regulate chondrocyte responses to mechanical stimuli, the molecular mechanisms underlying their synergy remain unclear. It is therefore recommended that future research focus on elucidating the pathways through which PIEZO1 and PIEZO2 interact at the molecular level, particularly in response to varying intensities of mechanical stress. A more profound comprehension of these mechanisms would facilitate our understanding of how these cells adapt to complex mechanical environments and could inform therapeutic strategies targeting Piezo channels in degenerative cartilage diseases.

### 6.3 L-Type Calcium Channels and Piezo1

L-type voltage-gated calcium channels (L-VDCCs) have been shown to play a critical role in mediating calcium influx during membrane depolarization in chondrocytes 102. These channels have been observed to interact with PIEZO1 to regulate calcium influx and exhibit synergistic effects in pathological responses of chondrocytes. Following the activation of PIEZO1 by abnormal mechanical stress, L-VDCCs have been shown to amplify calcium signaling, further increasing intracellular calcium concentrations 36,103.

In OA, PIEZO1 and L-VDCCs work collaboratively to enhance intracellular calcium signaling, a process which is associated with excessive mechanical stress and prolonged joint loading, both of which can exacerbate the progression of OA. L-VDCCs amplify calcium influx through PIEZO1, intensifying calcium signalling triggered by mechanical stimuli and worsening disease outcomes 104 Experiments using L-VDCC inhibitors, such as verapamil, demonstrate that blocking L-VDCCs significantly reduces high-strain-induced calcium influx influx, thus highlighting the supportive role of L-VDCCs in amplifying PIEZO1-mediated calcium signaling 105. Such synergistic effects are likely to magnify the response of chondrocytes to mechanical stress, influencing both physiological and pathological functions. In spinal growth plate chondrocytes, PIEZO1 and L-VDCCs synergistically amplify calcium signaling under compressive stress 42. However, whether this synergy exists in articular chondrocytes requires further validation. PIEZO1 and TRPV4 exhibit complementary roles: TRPV4 initiates low-intensity Ca²⁺ transients that prime PIEZO1 for high-strain responses via cytoskeletal prestress 20. Co-activation amplites ERK1/2 and YAP signaling, driving hypertrophic differentiation in OA 78. L-type Ca²⁺ channels further exacerbate Ca²⁺ overload by depolarizing the membrane, creating a voltage-PIEZO1 positive feedback loop 42. Dual inhibition of PIEZO1 and TRPV4/L-type channels may thus offer synergistic cartilage protection. However, the precise molecular mechanisms underlying this interaction remain to be elucidated. The following key questions require elucidation:

• Do PIEZO1 and L-VDCCs exhibit feedback mechanisms? PIEZO1-induced calcium influx might influence L-VDCC activity, creating a positive feedback loop that further elevates intracellular calcium levels. This could exacerbate stress-induced damage in OA.

• PIEZO1 and L-VDCCs share regulatory pathways: The investigation of shared signaling cascades has the potential to reveal new targets for the modulation of their activity in disease contexts.

• Differential roles in mechanical sensitivity exist: L-VDCCs might contribute more significantly to sustained calcium signaling, while PIEZO1 primarily mediates the initial mechanical response.

## 7. Conclusions and Perspectives

PIEZO1, as validated in chondrocyte-specific knockout models, is a critical regulator of articular chondrocyte mechanotransduction in OA, playing a central role in mechanical responses, matrix metabolism regulation, and inflammatory processes. As a mechanosensitive channel, PIEZO1 is essential for maintaining chondrocyte health and protecting articular cartilage. This review focuses on PIEZO1's roles explicitly validated in chondrocyte models. The regulation of PIEZO1 activation has significant clinical potential for the prevention of cartilage degeneration, the promotion of tissue repair, and the mitigation of the progression of joint diseases such as OA. Despite significant advancements in our understanding of PIEZO1's functions and mechanisms in chondrocytes, there are still substantial knowledge gaps, particularly concerning its activation dynamics, interactions with other channels, and therapeutic applications.

PIEZO1 activity correlates with OA severity, serving as a potential diagnostic biomarker via synovial fluid analysis. Pharmacologically, inhibitors like GsMTx4 reduce cartilage degradation in preclinical models, while activators (e.g., Yoda1) aid fracture repair under mechanical unloading. Natural compounds (e.g., artemisinin) suppress PIEZO1 overactivation, offering therapeutic potential. The spatiotemporal dynamics of PIEZO1 activation under various mechanical stresses, such as compression and stretching, and its behaviour in different cellular states, are not yet fully understood. Furthermore, further investigation is required into PIEZO1's interactions with other mechanosensitive channels, such as TRPV4 and PIEZO2. These channels appear to cooperate in regulating mechanical signaling, but the precise mechanisms of their synergy, particularly in pathological contexts such as joint injuries and OA, remain unclear. Furthermore, while PIEZO1 modulators, including activators and inhibitors, have demonstrated efficacy in preclinical studies, concerns regarding their specificity, selectivity, and potential adverse effects persist. The majority of available modulators are still in the preclinical stage and require extensive clinical validation.

The potential of PIEZO1 as a therapeutic target for the treatment of degenerative joint disease is a subject that offers considerable promise. Nevertheless, the successful translation of this research into clinical practice is predicated on the successful resolution of several significant challenges:

1. Molecular regulation: Future studies should focus on elucidating the molecular mechanisms underlying PIEZO1 activation, its regulatory networks and its interactions with other channels.

2. Channel synergy: Further research is required into the cooperative functions of PIEZO1 with TRPV4, PIEZO2 and other mechanosensitive channels under different mechanical stimuli. This will provide insights into their collective roles in cartilage physiology and pathology.

3. Finally, the development of new drugs should be a priority. Efforts should be directed towards the development of highly selective and potent PIEZO1 modulators (activators and inhibitors) and the evaluation of their safety and efficacy in both preclinical and clinical settings.

4. Combination therapies: Exploration of the synergistic potential of PIEZO1 inhibitors in combination with anti-inflammatory drugs or other agents could enhance therapeutic efficacy and minimise cartilage degeneration.

The present study investigates the role of PIEZO1 in cartilage biology and disease progression, thereby highlighting its potential as a transformative target for understanding and treating cartilage degeneration, repair, and development. The multifactorial regulation of PIEZO1 not only deepens our understanding of OA pathophysiology but also opens avenues for clinical innovation. Targeting PIEZO1 hyperactivity with inhibitors like GsMTx4 or natural compounds (e.g., artemisinin) offers potential disease-modifying therapies, while its context-dependent activation may aid cartilage repair. Advancing research into its molecular regulation, synergistic interactions, and pharmacological modulation will provide valuable insights and therapeutic strategies, ultimately benefiting patients suffering from OA and other degenerative joint disorders.

## Figures and Tables

**Figure 1:**
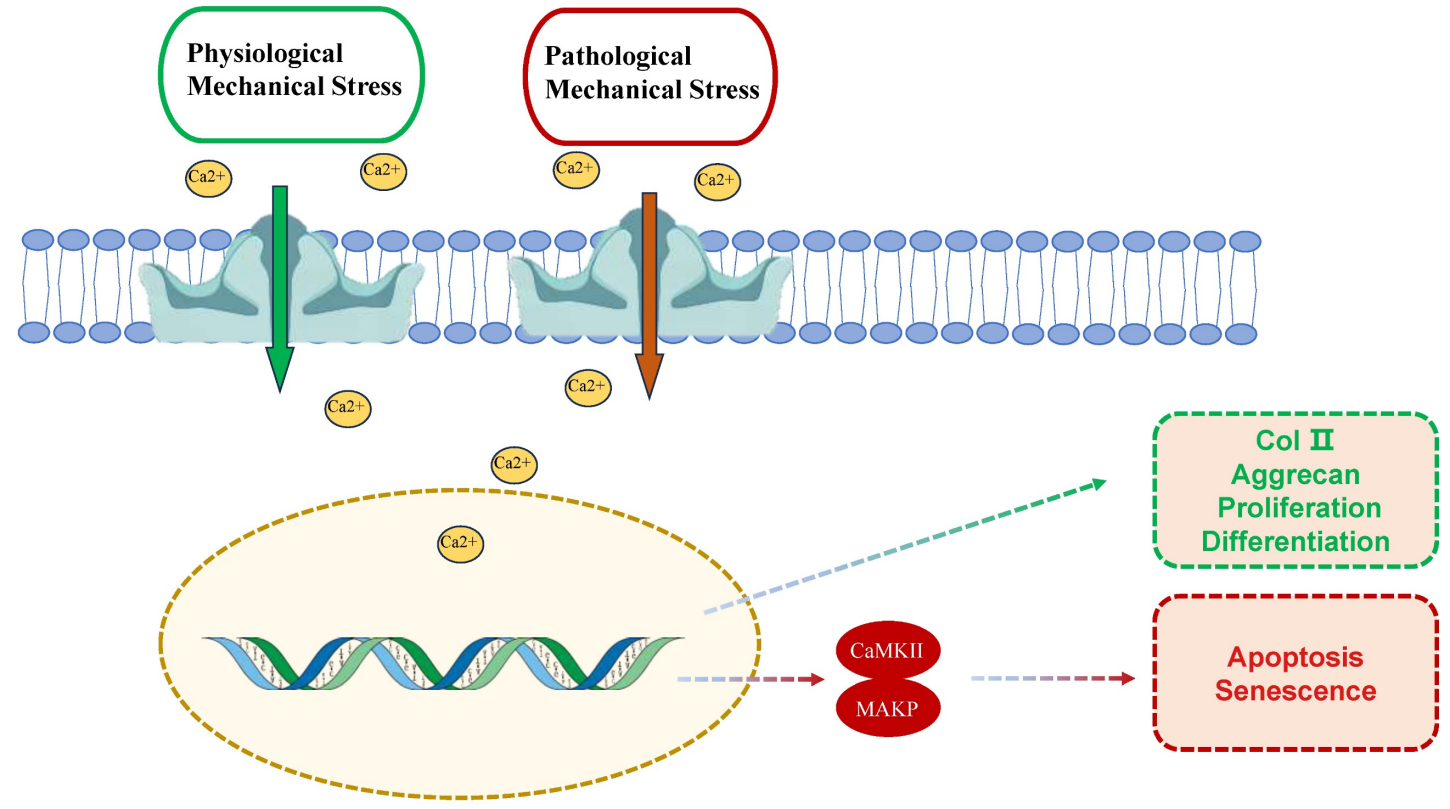
The dual role of PIEZO1 activation in chondrocytes under physiological and pathological mechanical stress. Under physiological conditions (green box), moderate mechanical stress activates PIEZO1, leading to calcium influx, which supports cellular functions such as ECM synthesis (e.g., type II collagen and aggrecan) and chondrocyte proliferation and differentiation. However, under pathological conditions (red box), excessive mechanical stress over activates PIEZO1, resulting in excessive calcium influx, apoptosis, and cellular senescence. This process involves activation of key intracellular signaling pathways, including CaMKII and MAPK, which exacerbate cartilage degeneration.

**Figure 2:**
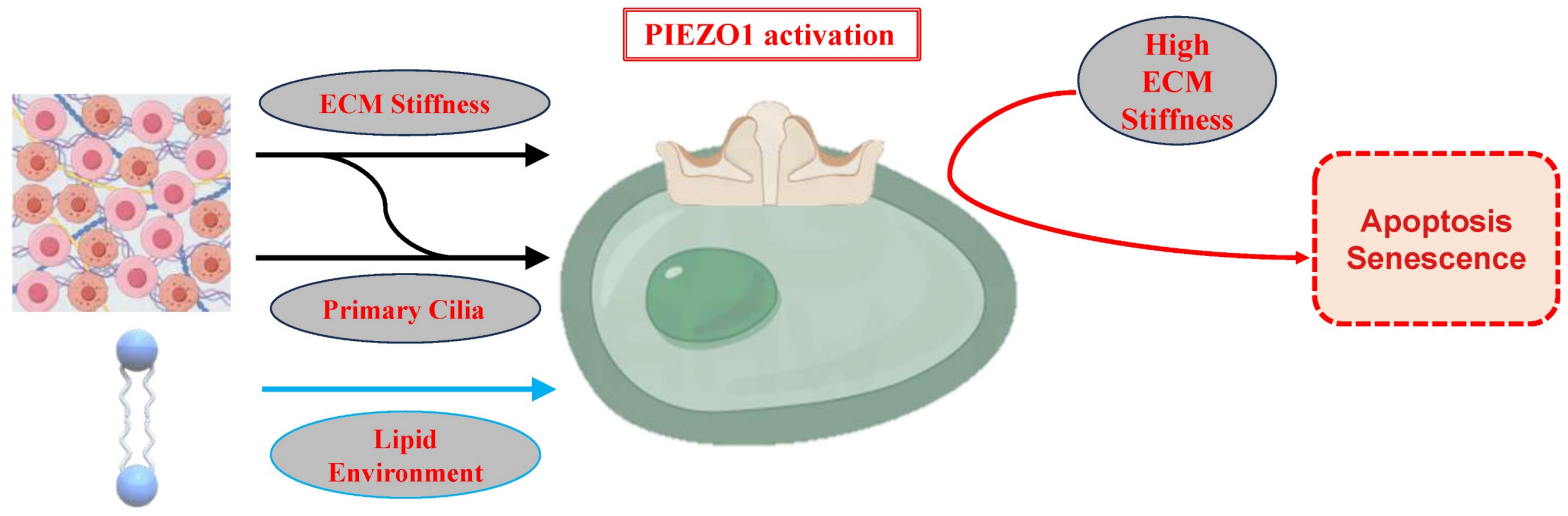
Regulation of PIEZO1 Activation by ECM Stiffness, Primary Cilia, and Lipid Environment. Low ECM stiffness inhibits PIEZO1, preserving chondrocyte phenotype and calcium homeostasis. High ECM stiffness activates PIEZO1, enhancing calcium influx and stress responses, contributing to senescence and apoptosis. Primary cilia modulate PIEZO1 sensitivity, while the lipid bilayer influences PIEZO1 activity.

**Figure 3:**
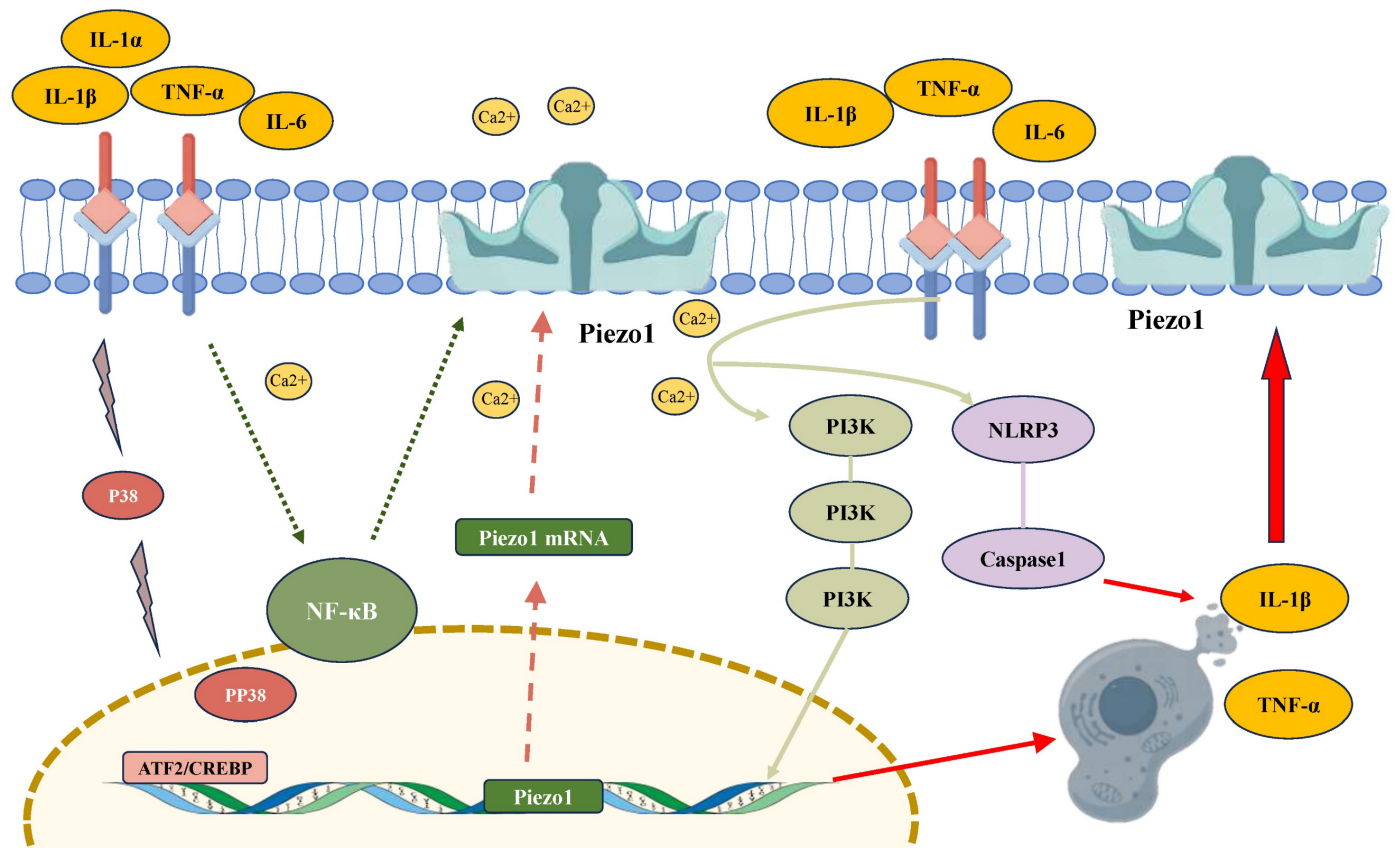
The regulatory role of inflammatory factors in modulating PIEZO1 activity in chondrocytes. Inflammatory cytokines, such as IL-1β and TNF-α, activate PIEZO1 channels, leading to increased intracellular calcium (Ca²⁺) influx. This activation upregulates PIEZO1 expression via signaling pathways involving NF-κB, PI3K, and ATF2/CREBP1. The enhanced PIEZO1 activity promotes inflammation by facilitating the release of pro-inflammatory cytokines (IL-1β, TNF-α), forming a feedback loop that exacerbates cellular damage. Furthermore, PIEZO1 activation triggers the NLRP3 inflammasome and caspase-1, contributing to the maturation of IL-1β and further promoting chondrocyte apoptosis.

**Figure 4:**
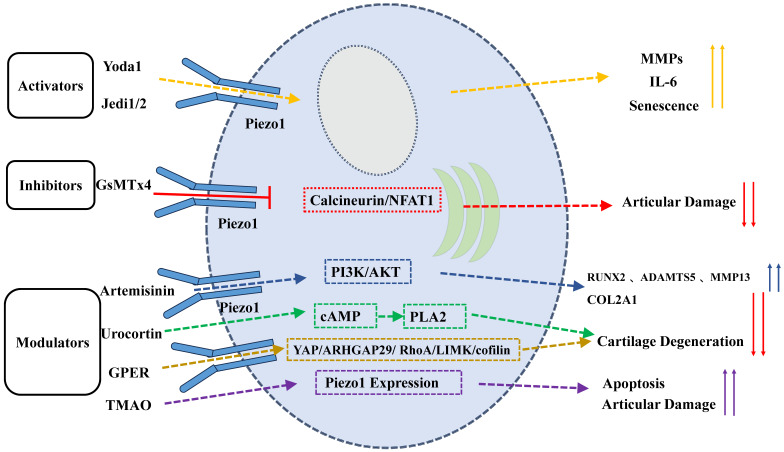
Modulation of PIEZO1 Activity by Activators, Inhibitors, and Modulators. Activators like Yoda1 and Jedi1/2 enhance PIEZO1 activity, promoting calcium influx, senescence, ECM degradation, and inflammation. Inhibitors like GsMTx4 prevent activation, preserving cartilage integrity. Natural compounds and modulators (e.g., artemisinin, urocortin, TMAO, GPER) regulate PIEZO1, affecting cartilage degeneration and apoptosis.

**Figure 5:**
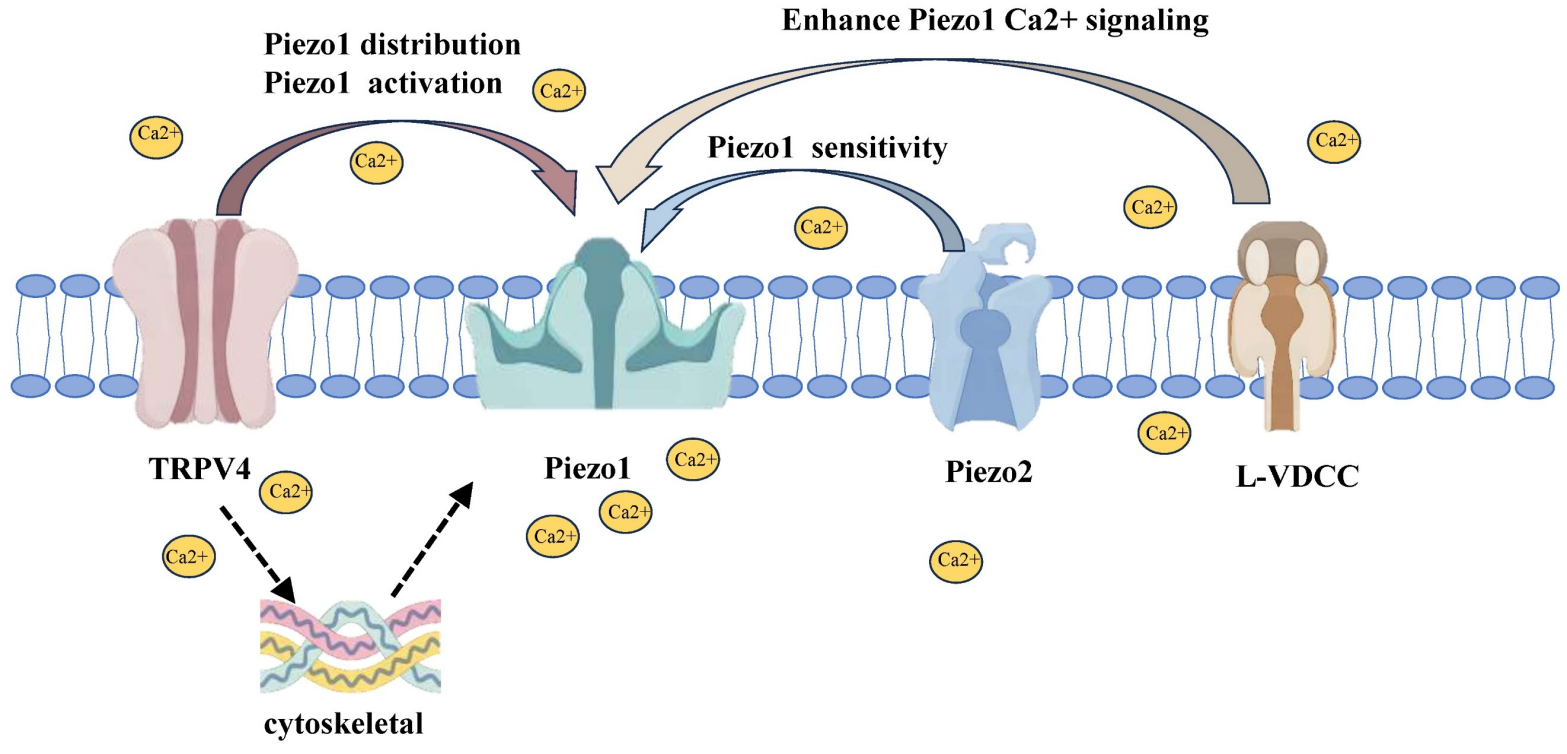
The interactions between PIEZO1 and other mechanosensitive channels. TRPV4 modulates PIEZO1 distribution and activation by responding to low-intensity mechanical stress, enhancing PIEZO1 activity and calcium signaling. PIEZO2, primarily involved in low-intensity stress, enhances PIEZO1 sensitivity, thereby facilitating more efficient responses to mechanical stimuli. L-VDCCs amplify PIEZO1-mediated calcium influx, intensifying calcium signaling, especially during high mechanical stress. Together, these channels form a coordinated network that regulates chondrocyte mechanotransduction.

**Figure 6:**
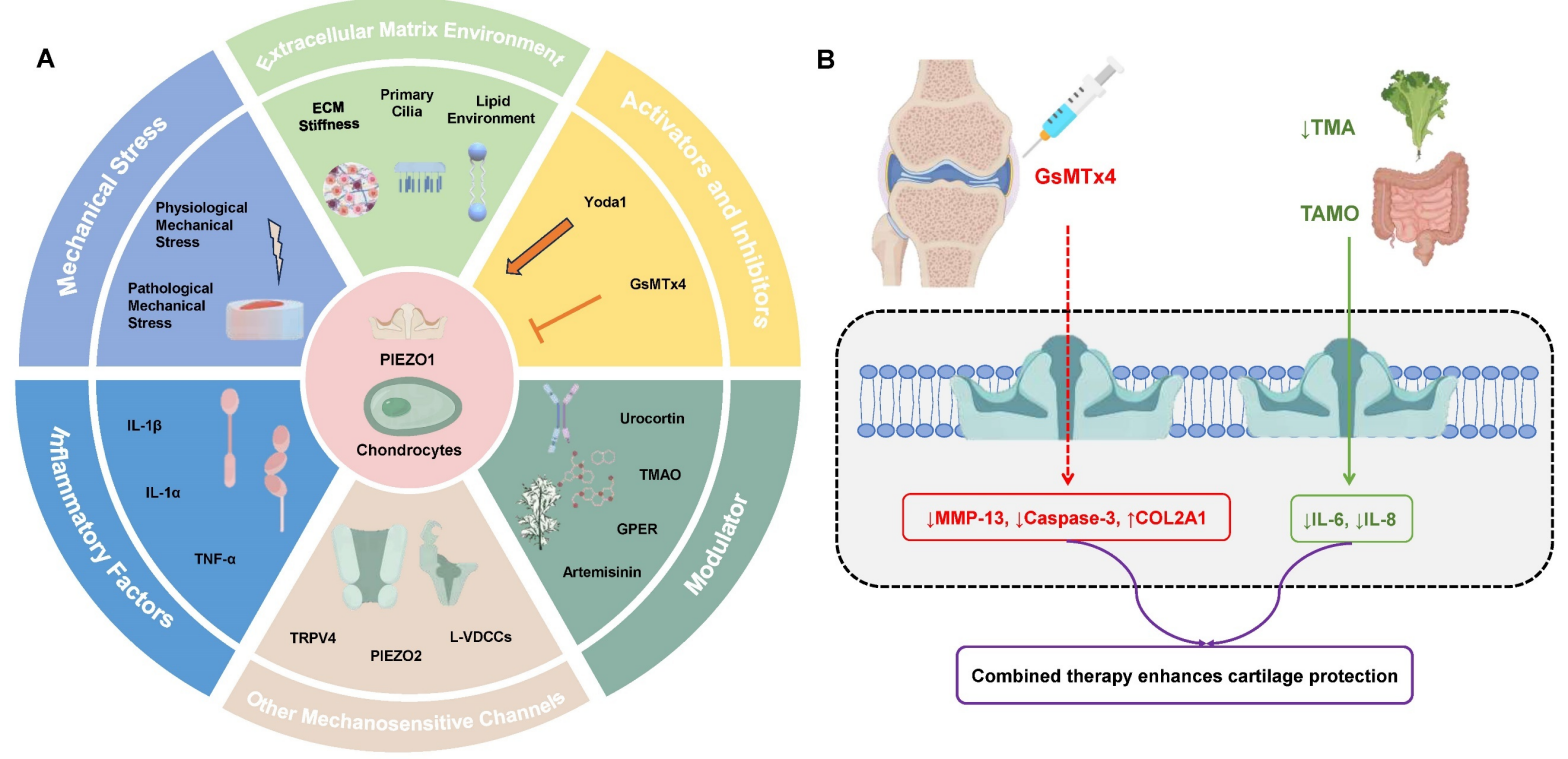
A: Overview of PIEZO1 Regulation in Chondrocytes. Mechanical Stress: Both physiological and pathological mechanical stress regulate PIEZO1 activation, impacting cartilage function and health. Physiological stress supports ECM synthesis, while pathological stress leads to excessive calcium influx, contributing to cartilage degradation. Extracellular Matrix (ECM) Environment: ECM stiffness and primary cilia significantly affect PIEZO1 activity. High ECM stiffness enhances PIEZO1 activation, while primary cilia modulate PIEZO1 sensitivity. Inflammatory Factors: Cytokines like IL-1β and TNF-α upregulate PIEZO1 expression and its activation, exacerbating cartilage degradation. Other Mechanosensitive Channels: PIEZO1 interacts with channels like TRPV4 and PIEZO2, enhancing calcium signaling and regulating cellular responses to mechanical stimuli. Activators and Inhibitors: Chemical agents such as Yoda1 and Jedi1/2 activate PIEZO1, while GsMTx4 inhibit its activity, offering potential therapeutic avenues for modulating PIEZO1 in OA. Modulators: Natural compounds like urocortin, TMAO, and artemisinin, along with the GPER receptor, also modulate PIEZO1 activation and its effects on chondrocyte functions. B: Clinical strategies targeting PIEZO1 in OA management: Intra-articular injection of the PIEZO1 inhibitor GsMTx4 blocks pathological Ca²⁺ influx, reducing MMP-13 expression and apoptosis while preserving ECM integrity. Dietary reduction of TMAO (e.g., low-meat/high-fiber diets) suppresses gut microbiota-derived TMAO, thereby downregulating PIEZO1 expression and mitigating cartilage degradation. Combined approaches may offer enhanced therapeutic efficacy by addressing both mechanical and inflammatory drivers of OA.

**Table 1:** Drugs that can regulate piezo1 in chondrocytes.

Category	Drug	Property	Selectivity	Experimental Model	Experiment	Mechanism of Action	References
Activator	Yoda1	Compound	Specific	Human chondrocytes	*In vitro*	Lowers the mechanical threshold for channel activation; increases the expression of pro-inflammatory cytokines IL-6 and IL-8, MMP1, MMP3, and MMP13, while decreasing BMP2 expression in healthy and OA cartilage cells.	Syeda et al., 2015 30
				Rat chondrocytes	*In vitro*	Ca²⁺ accumulation decreases chondrocyte proliferation, induces cellular senescence, increases markers p16 and p21, and raises SASP markers IL-6 and MMP3.	Ren et al., 2022 72
				Mice	*In vivo*	Increases intracellular calcium influx, influences PI3K/AKT phosphorylation; intra-articular injection worsens OA pathology (cartilage erosion, proteoglycan loss, osteophyte growth, and pain); OA-related genes RUNX2, MMP13 are upregulated, COL2A1 is downregulated.	Gan et al., 2024 83
				Human OA chondrocytes	*In vitro*		
				ATDC5 cells	*In vitro*	Yoda1 induces a twofold increase in Sox9 and enhances the expression of *Ccn2/Ctgf* genes.	Brylka et al., 2024 21
Inhibitor	GsMTx4	Polypeptide toxin	Non-specific	Rat and chondrocytes	*In vivo* and *in vitro*	Intra-articular injection improves OA progression by reducing MMP3 and MMP13 expression and increasing COL2 and Aggrecan expression; blocks Piezo1/CaN/NFAT1 signaling to inhibit apoptosis and promote cartilage matrix production.	Ren et al., 2023 32
				Mice	*In vivo*	Intra-articular injection increases Gpx4 expression, alleviates ferroptosis, and reduces OA severity in surgery-induced models.	Wang et al., 2022 79
				Pig joint organ culture	*In vitro*	Preventive use reduces mechanically induced damage regions.	Lee et al., 2014 78
				Pig chondrocytes	*In vitro*	GsMTx4 prevents inflammation-induced F-actin disassembly or relaxation, maintaining membrane elasticity under IL-1α.	Lee et al., 2017 36
Modulators	Urocortin-1	Neuropeptide	Non-specific	Human chondrocytes	*In vitro*	Binds to CRF-R1, activates adenylyl cyclase to generate cAMP, leading to PLA2 inactivation, reducing membrane phospholipid degradation, and keeping Piezo1 closed. Stabilizes membrane phospholipids	Lawrence et al., 2017 84; Jones et al., 2022 85;
				Pig joint organ culture	*In vitro*		
	GPER	Membrane receptor protein	Non-specific	Rat chondrocytes	*In vivo* and *in vitro*	Leads to actin depolymerization via YAP/ARHGAP29/RhoA/LIMK/cofilin pathway; blocks Piezo1 activation by mechanical stress, improving chondrocyte survival.	Sun Y et al., 2021 38​
	TMAO	Organic compound	Non-specific	Rats	*In vivo* and *in vitro*	Intra-articular injection and *in vitro* studies show TMAO increases Piezo1 expression, raises baseline Ca²⁺ levels, reduces F-actin, and increases apoptosis under mechanical strain.	Zhuang et al., 2023 88​
	Artemisinin	Traditional medicine extract	Non-specific	Mice	*In vivo*	Inhibits calcium influx induced by Piezo1 activation, modulates PI3K/AKT phosphorylation, reverses OA-related gene expression, and mitigates Piezo1 activation-induced OA damage.	Gan et al., 2024 83

**Table 2:** Evidence hierarchy for piezo1 functions in chondrocytes.

Tier	Study Type	Example References	Experimental Model	Support Strength for Chondrocyte Conclusions
Tier 1: Chondrocyte-Specific Models	Primary chondrocytes, chondrocyte-targeted genetically modified mice	Brylka et al., 2024 (Ref 21)	*Col2a1-Cre; Piezo1ᶠˡ/ᶠˡ* mice	★★★★★
		Sun L et al., 2025 (Ref 69)	*Col2a1^CreERT^; Piezo1^flox/flox^* mice	★★★★★
		Sun Y et al., 2021 (Ref 38)​	Human OA chondrocytes + murine joint injury model	★★★★★
		Savadipour et al., 2023 (Ref 47)	Primary chondrocyte compression assays	★★★★☆
Tier 2: Non-Specific *In vivo* Models	Systemic knockout mice, pharmacological intervention models	Lee et al., 2014 (Ref 78)	Global *Piezo1⁻/⁻* mice	★★☆☆☆
		Gan et al., 2024 (Ref 83)	DMM model mice + artemisinin intervention	★★★☆☆
Tier 3: Non-Chondrocyte Models	Endothelial cells, neurons, other cell lines	Coste et al., 2010 (Ref 12)	Mechanosensitivity studies in neuronal cells	★☆☆☆☆
		Syeda et al., 2015 (Ref 30)	HEK293 cells + Yoda1 activation mechanism	★☆☆☆☆

Key Notes: Tier 1: Direct evidence from chondrocyte-specific models (highest reliability). Tier 2: Systemic or non-specific models (caution required for cartilage-specific conclusions). Tier 3: Findings from non-chondrocyte systems (exploratory only, not generalizable to chondrocytes). Star Rating: ★★★★★ = Strongest evidence; ★☆☆☆☆ = Weakest evidence.

**Table 3:** Summary of mouse model data in piezo1 regulation studies.

Category	Mouse Model Type	Experiment (*In vivo*/*In vitro*)	Effect	Limitations	References
Mechanical Stress	Global *Piezo1* knockout (*Piezo1⁻/⁻*)	*In vivo*	High mechanical stress induces chondrocyte apoptosis and accelerates OA progression.	Systemic knockout affects other tissues; cartilage-specific effects cannot be isolated.	Lee et al., 2014 78
	Chondrocyte-specific *Piezo1* knockout (*Col2a1-Cre; Piezo1ᶠˡ/ᶠˡ*)	*In vivo*	Loss of *Piezo1* in chondrocytes impairs endochondral ossification and reduces OA severity.	Limited to developmental stages; adult OA-specific effects require further validation.	Brylka et al., 2024 21
	Primary chondrocytes under compression (15% strain)	*In vitro*	PIEZO1 activation triggers Ca²⁺ influx and ECM degradation.	*In vitro* models lack systemic inflammatory or biomechanical context.	Savadipour et al., 2023 47
ECM Environment	3D hydrogel culture (stiffness gradient)	*In vitro*	High stiffness (>20 kPa) upregulates PIEZO1 expression and promotes senescence.	Hydrogel stiffness may not fully replicate native cartilage ECM complexity.	Du G et al., 2022 37
	Primary cilia-deficient mice (*Ift88⁻/⁻*)	*In vivo*	Impaired ECM stiffness sensing and altered PIEZO1 activation thresholds.	Global cilia dysfunction affects multiple cell types beyond chondrocytes.	Guo et al., 2024 63
Inflammatory Factors	IL-1β intra-articular injection (C57BL/6 mice)	*In vivo*	IL-1β upregulates PIEZO1 via NF-κB, exacerbating cartilage degradation.	Acute inflammation model may not mimic chronic OA progression.	Lee et al., 2021 48
	TNF-α stimulation in primary chondrocytes	*In vitro*	TNF-α enhances PIEZO1 open probability via ROS-mediated oxidation.	*In vitro* ROS levels may exceed physiological relevance.	Lee et al., 2021 48
	Chondrocyte-specific Piezo1 activation under stress	*In vivo* + *In vitro*	Mechanical overload → Piezo1-mediated mtDNA release → activates cGAS-STING → inflammation via IFN-β, IL-6, TNF-α.	Model focuses on acute inflammatory amplification; long-term OA outcomes need validation.	Sun L et al., 2025 69
Agonists	Intra-articular Yoda1 (wild-type mice)	*In vivo*	Yoda1 activates PIEZO1, increasing senescence and MMP-13 expression.	Off-target effects of Yoda1 on other ion channels are not ruled out.	Gan et al., 2024 83
Inhibitors	Intra-articular GsMTx4 (surgery-induced OA mice)	*In vivo*	GsMTx4 inhibits PIEZO1, reducing cartilage erosion and pain.	Peptide-based inhibitors face challenges in clinical translation (e.g., stability, delivery).	Ren et al., 2023 32
Modulators	Artemisinin dietary intervention (DMM model mice)	*In vivo*	Suppresses PIEZO1 activity, improving cartilage integrity.	Low bioavailability of oral artemisinin in joints may limit efficacy.	Gan et al., 2024 [83
	Urocortin-treated cartilage explants	*In vitro*	Inhibits PIEZO1 via CRF-R1/cAMP signaling, reducing mechanical injury.	Explant models lack dynamic mechanical loading and systemic feedback.	Lawrence et al., 2017 84
Other Ion Channels	*TRPV4/PIEZO1* double knockout (*Col2a1-Cre* mice)	*In vivo*	Dual knockout suppresses mechano-induced Ca²⁺ signaling, protecting cartilage.	Compensatory upregulation of other mechanosensitive channels (e.g., TRPA1) not assessed.	Servin-Vences et al., 2017 20
	L-type Ca²⁺ channel inhibitor (Verapamil in OA mice)	*In vivo*	Reduces PIEZO1-mediated Ca²⁺ overload, alleviating OA.	Verapamil's cardiovascular side effects complicate systemic use for OA.	Takamatsu et al., 2023 104
